# Technology Assistance in Dementia (Tech‐AiD): Leveraging Everyday Technologies to Support Dementia Care

**DOI:** 10.1002/alz.095390

**Published:** 2025-01-09

**Authors:** Andrew M Kiselica

**Affiliations:** ^1^ University of Missouri, Columbia, MO USA

## Abstract

**Background:**

Care partners to persons with dementia increasingly use everyday technologies, like smartphones, smart speakers, and wearable device, to improve care and minimize burden. However, there remains a significant gap in effective implementation of these devices in care routines. For example, while as many 90% of care partners own smartphones, less than 50% use these devices on a daily basis, and less than 30% take advantage of their assistive features. As a result, functions that may aid in caring, like GPS for navigation, notes for to‐do lists, and alarm reminders for appointments, are not routinely employed. Thus, there is a need for interventions to improve effective digital technology use among care partners to persons with dementia. This presentation will describe progress in developing a novel program to meet this need, called Technology Assistance in Dementia (Tech‐AiD).

**Methods:**

The presenter will discuss results from four Stage I behavioral intervention development studies, including a qualitative interview study of 22 care partners to better understand technology use, a survey study of 100 care partners to develop improved measures of technology‐based strategies, development of an intervention framework, and a mixed methods study among 10 care partners and 10 healthcare professionals to gather feedback on the intervention.

**Results:**

An initial qualitative research study highlighted six domains of technology use in dementia care and also brought to light drawbacks of technology use, including cost, complexity, and privacy concerns. Next, the survey resulted in the development of the Technology in Caring Questionnaire (TCQ) to capture technology‐based strategy use. These studies informed the development of a treatment framework for Tech‐AiD, which was rated as appropriate, feasible, and acceptable by care partners and healthcare professionals.

**Conclusions:**

The findings set the stage for a Stage IB pilot randomized control trial of Tech‐AiD to evaluate its feasibility, acceptability, and preliminary efficacy.

